# A novel natural product inspired scaffold with robust neurotrophic, neurogenic and neuroprotective action

**DOI:** 10.1038/srep14134

**Published:** 2015-09-21

**Authors:** Sumana Chakravarty, Swati Maitra, R Gajendra Reddy, Tapatee Das, Priya Jhelum, Scherazad Kootar, Wenson D. Rajan, Anumita Samanta, Ramesh Samineni, Srihari Pabbaraja, Steven G. Kernie, Goverdhan Mehta, Arvind Kumar

**Affiliations:** 1 Chemical Biology, CSIR- Indian Institute of Chemical Technology, Tarnaka, Uppal Road, Hyderabad 500007, India; 2 CSIR- Centre for Cellular and Molecular Biology, Habsiguda, Uppal Road, Hyderabad 500007, India; 3 Natural Products Chemistry, CSIR- Indian Institute of Chemical Technology, Tarnaka, Uppal Road, Hyderabad 500007, India; 4 Department of Pediatrics and Department of Pathology and Cell Biology, College of Physicians and Surgeons, Columbia University, New York, NY 10032, USA.; 5 School of Chemistry, University of Hyderabad, Hyderabad; 500046, India

## Abstract

In search for drugs to treat neuropsychiatric disorders wherein neurotrophic and neurogenic properties are affected, two neurotrophically active small molecules specially crafted following natural product leads based on 2-oxa-spiro[5.5]-undecane scaffold, have been thoroughly evaluated for their neurotrophic, neurogenic and neuroprotective potential in *ex vivo* primary culture and *in vivo* zebrafish and mouse models. The outcome of *in vivo* investigations suggest that one of these molecules is more neurotrophic than neurogenic while the other one is more neurogenic than neurotrophic and the former exhibits remarkable neuroprotection in a mouse acute ischemic stroke model. The molecular mechanisms of action of these compounds appear to be through the TrkB-MEK-ERK-CREB-BDNF pathway as pre-treatment with neurotrophin receptor TrkB inhibitor ANA-12 and MEK inhibitor PD98059 attenuates the neurotrophic action of compounds.

The nature of brain and behavior disorders, including cognitive impairments, unfolds differently during the lifespan of individuals. The young ones are afflicted with higher incidence of psychiatric disorders such as depression, anxiety, schizophrenia, substance abuse, whereas the elders appear to be more vulnerable to neurodegenerative diseases like various forms of dementia, stroke, and etc^1^. There is marked surge in the geriatric population worldwide and consequently an alarming rise in the neurodegenerative central nervous system (CNS) disorders, broadly categorized as ‘dementia’ (i.e. cognitive diminution affecting learning and memory), which includes Alzheimer’s disease and cerebral stroke-induced vascular dementia. The neuropathological studies show that most of these disorders are associated with attenuated neurotrophic activities and/or neurogenesis^2^. However, there is dearth of therapeutic interventions that can slow down neurodegeneration and help in the repair and regeneration of neurons in the affected brain^3^. In order to help the patients of dementia or stroke to recover fast from these debilitating conditions, a potential drug should have the ability to promote neurite growth and synaptic plasticity through enhanced neurotrophic support by augmenting and sustaining the levels of endogenous neurotrophins [such as Nerve Growth Factor (NGF), Brain Derived Neurotrophic Factor (BDNF), Glial cell-derived neurotrophic factor (GDNF), Neurotrophin-3 (NT3), Neurotrophin-4 (NT4), etc.] and/or increasing the adult neurogenesis in the affected circuitry. In recent years, a few small molecule natural compounds have been shown to promote neurite growth and synaptic plasticity by up-regulating the activity of neurotrophins^4^,^5^. However, these efforts are in early stages and have not advanced towards establishing the therapeutic efficacy to decelerate neurodegeneration and boost neurogenesis, in order to treat cognitive disorders. Thus, the search for novel chemical entities with neurotrophic and/or neurogenic action to treat CNS disorders is being actively pursued^6^. In this context, natural product leads from cell-based assays provide a promising platform to build upon. Recently, we have reported the crafting of a novel 2-oxa-spiro[5.5]-undecane scaffold inspired by the natural product Paecilomycine A, following the tactic of diverted organic synthesis (DOS)^7^. Based on significant neurotrophic activity in cell-based assays^7^ exhibited by this scaffold and partial mapping of the chemical space around it, two tricyclic molecules, compound #1 and #2 (Fig. 1) were identified for further evaluation of their neurotrophic, neurogenic as well as neuroprotective ability in the *in vivo* system, i.e. in vertebrate brain. In this quest, diverse models such as *in vitro* cell lines, *ex vivo* mouse primary neurosphere and brain slice cultures and *in vivo* zebrafish and mouse brain were used and efforts were made to identify the mechanisms of action at the cellular and molecular level.

## Results

### Structure of compound 1 (comp#1) and compound 2 (comp#2)

**Figure 1:**
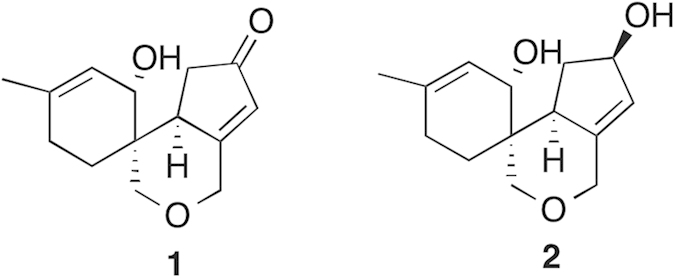
Structures of compound#1 and compound#2.

The lead molecules in this study (shown in Fig. 1) that we tested for their neuroactivity in *ex vivo* and *in vivo* models, were synthesized through a concise strategy as described in detail previously^7^.

### Evaluation of the neurogenic potential of comp#1 and comp#2 in *ex vivo* neurosphere assay

**Figure 2:**
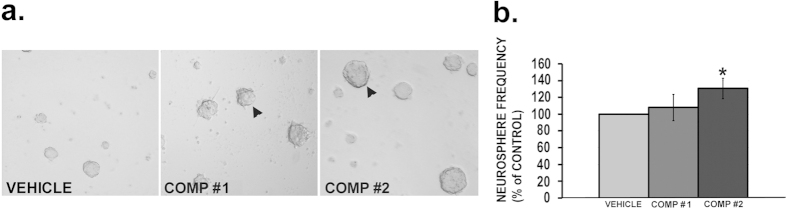
Neurosphere assay: By using neural stem/precursor cell population from postnatal (P2-3) mouse hippocampus (a–b). Results are expressed as mean ± SEM where neurosphere frequency (>100 μm size) represented as percentage of control (n = 3/group); *p < 0.05 compared with control. (Student’s T-test).

Once we could reproduce the potent neurotrophic activity of the new batch of comp#1 and comp#2 as reported earlier^7^ by us in the preliminary screening on Neuro2A cell line, studies were initiated to find out the neurogenic potential of these compounds. Neonatal (postnatal day 2) mouse neural stem cells or neural progenitor cells (NSCs/NPCs) from hippocampal dentate gyrus (DG) in culture were treated with comp#1 (0.01 μM), comp#2 (0.01 μM) and vehicle (1% DMSO). The dose (0.01 μM) used was proneurogenic in the preliminary *ex vivo* neurosphere assay (Supplementary Fig. 1). The treatment of NSCs/NPCs with comp#2, but not comp#1, showed robust increase (approximately 30%) in the number of larger neurospheres measuring >100 μm compared to the ones treated with the vehicle (*p < 0.05), which is suggestive of good neurogenic potential of comp#2 (Fig. 2a,b).

### Evaluation of the neurogenic activity of comp#1 and comp#2 in Zebrafish and Mouse brain

**Figure 3:**
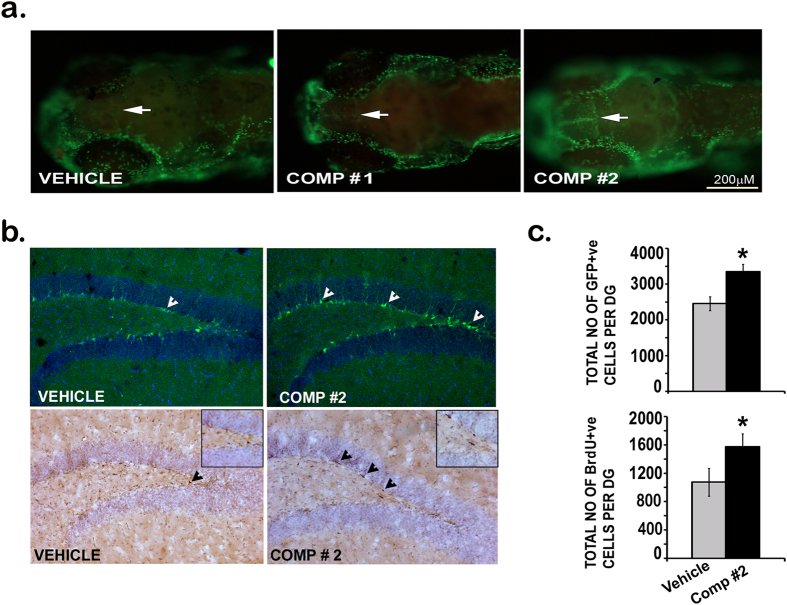
Neurogenic activity of compounds in *In vivo* (Zebrafish, Nestin-GFP mice). Whole mount immunofluorescence for BrdU in 4dpf zebrafish brain (**a**) Comp#1 and comp#2 both induce neurogenesis as shown by the induction of the beginning of telencephalon (**a**) Shown are representative photomicrographs of Nestin-positive cells (top panel) and BrdU-positive cells (bottom panel) (**b**) from vehicle (DMSO 1%) and compound -treated groups. Number of Nestin-positive and BrdU-positive cells in the SGZ at the border of the GCL (n = 4-5/group) *p < 0.05 (**b**,**c**).

The differential neurogenic activity of comp#1 and comp#2 in primary neural stem/progenitor cell cultures led us to further investigate the efficacy of these small molecules *in vivo,* in vertebrate brain, after crossing the blood brain barrier (BBB) in zebrafish larvae. At 3 day post fertilization (dpf) when the BBB gets fully established in zebrafish larvae, the larvae were treated with vehicle (1% DMSO) and 1 μM of compounds #1 and #2, the dose found to be safe and non-toxic in initial study on embryos (Supplementary Fig. 1). After 24 h of the treatment, 4 dpf larvae were incubated with the cell proliferation marker BrdU and immunostained using anti-BrdU antibody. The images of BrdU labeled larvae were captured by microscope and the analysis showed robust increase in the population of BrdU+cells in the telencephalic region, the beginning of the sub-ventricular zone (SVZ) in Zebrafish, in comp#2 treatment (Fig. 3a). Comp#1 treatment showed only slight increase in neurogenesis when compared to the vehicle-treated larvae (Fig. 3a).

The impressive proneurogenic activity of comp#2 in zebrafish larval brain led us to further explore its neurogenic potential in a higher vertebrate, the mouse. Nestin GFP reporter mice were used where all NSCs/NPCs in neurogenic niches in the adult brain, including the hippocampal dentate gyrus (DG), express GFP (green fluorescent protein) under nestin promoter. Nestin GFP mice in two groups were injected comp#2 (1 mg/kg) and vehicle for 7 consecutive days, followed by daily injection of BrdU (50 mg/kg) 30 min after the administration of compound and the vehicle. The immunohistochemistry was done on sliced mouse brains and the quantitative analysis of Nestin-GFP + ve and BrdU + ve cells in the neurogenic dentate gyrus of mouse hippocampus suggests that comp#2 indeed has remarkably increased the turnover of NSCs/NPCs as compared to the vehicle-treated mice (*p < 0.05), in both fluorescence based Nestin-GFP count (the upper panel of Fig. 3b,c) and non-fluorescent immunohistochemistry based BrdU count (the lower panel of Fig. 3b,c), respectively.

### Both compounds activate the expression of a number of neuroactive genes and proteins

**Figure 4:**
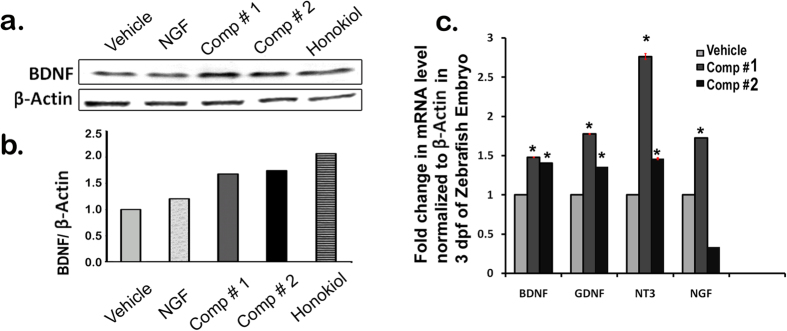
Immunoblot and q-PCR data for compound induced neurotrophins expression. Immunoblot data showing BDNF expression in Neuro2A cells upon treatment of Vehicle (1% DMSO), NGF (200 ng/ml), comp#1 (0.01 μM), comp#2 (0.01 μM), Honokiol (1 μM) (a–b). Relative gene expression level of the neurotrophic factors like BDNF, GDNF, NT3 and NGF in zebrafish larvae. Values are means ± SEM, (n = 3) and each group containing 60 larvae (**c**) *p < 0.005.

To assess the neurotrophic and neurogenic action of both the compounds at molecular level, the pool of 3dpf larvae were treated with compounds (1 µM) and vehicle and change in the level of mRNA for genes that code for neurotrophic and neurogenic factors such as BDNF, GDNF, NT3 and NGF was analyzed. Comp#1 and comp#2 showed substantial increases in the expression of BDNF, GDNF and NT3, compared to the level seen in vehicle-treated embryos (shown in Fig. 4c). However, while the level of NGF transcripts were high in larvae treated with comp#1, comp#2-treated larvae showed significantly lower NGF mRNA level compared to the vehicle (1% DMSO) treatment (Fig. 4c).

Brain derived neurotrophic factor (BDNF) is the most widely expressed neurotrophic factor in brain and plays critical role in the development and functioning of neurons, neural circuits and synaptic plasticity^1^,^6^,^8^. So, the compounds were further evaluated in Neuro2A cell line for their role in the induction of BDNF protein. Comp#1 (0.01 μM) and comp#2 (0.01 μM) both were potent in increasing the BDNF transcript level, similar to the level induced by the positive control honokiol (1 μM) (Fig. 4a,b).

### Neuroprotective efficacy of comp#1 and comp#2 in mouse ischemic stroke model

**Figure 5:**
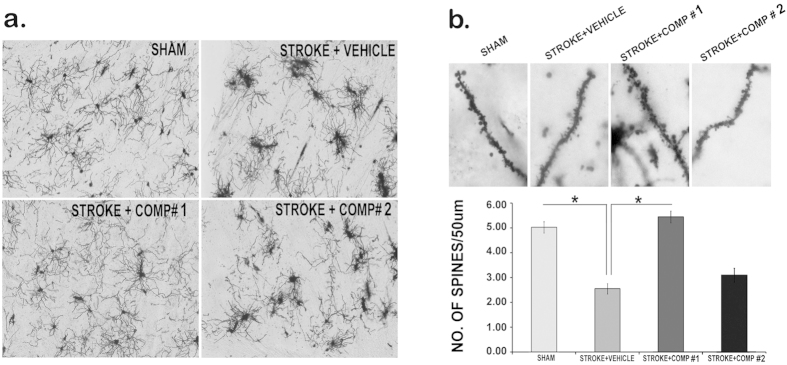
Neuroprotection of compounds in Ischemic stroke model (Mice). BCCAO induced impairment in synaptic connectivity in the striatal region of mouse brain (see in stroke + vehicle group), compared to sham control, using Golgi cox staining (a) intact synaptic connections observed in comp#1 (10 mg/kg)-treated brain (stroke + comp#1). Note improvement in spine density with comp#1 (10 mg/kg) treatment to the level similar in sham control (no stroke group) *p < 0.05 (b).

Having observed that comp#2 is proneurogenic *in vivo*, while both comp#1 and #2 are neurotrophic in cell (Neuro2A) based assays, it was of interest to evaluate their neuroprotective potential against acute ischemic insult in bilateral common carotid artery occlusion (BCCAO)-induced global ischemia mouse model wherein the neurotrophic and neural repair or regeneration are compromised. Mice were pre-treated with comp#1 (10 mg/kg), comp#2 (10 mg/kg) and the vehicle (1% DMSO) 15 min before the BCCAO-induced global ischemia. 24 hrs post-ischemic insult the integrity of the neural connectivity was analyzed using Golgi-Cox method and the result suggested robust neuroprotection by comp#1, as evident by the almost intact neural connections similar to what was observed in SHAM control (Fig. 5a); in contrast, in mice subjected to BCCAO the pretreatment with comp#2 failed to provide neuroprotection against the ischemic insult and the neural connections were severely disrupted in the affected striatal region, as also shown by mice pretreated with the vehicle (Fig. 5a). Similar result was observed upon the analysis of spine numbers in these groups: in contrast to the stroke + vehicle group, the stroke + comp#1 group exhibited augmented spine numbers while stroke + comp#2 groups showed no improvement similar to stroke + vehicle group (Fig. 5b).

### The neurotrophic property of comp#1 and comp#2 in Neuro2A cell line and Zebrafish is mediated via MEK-ERK Pathway

**Figure 6:**
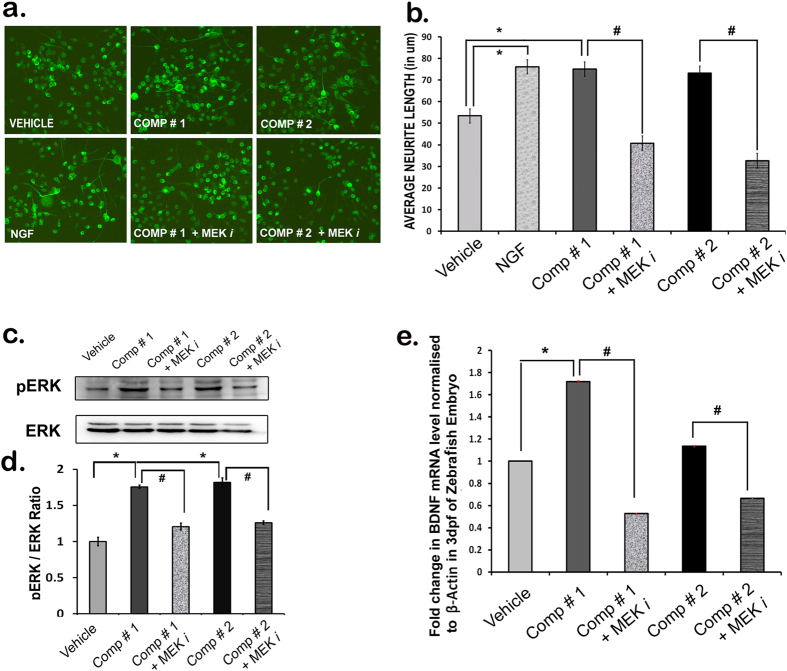
Mechanistic studies of compounds with MEK inhibitor *in vitro* and *in vivo*. Morphometric analysis of neurite outgrowth of differentiated Neuro2a cells treated with comp#1 (0.01 μM), comp#2 (0.01 μM), pre-treated (with or without MEK inhibitor PD98059) (20 μM) (a–b) expression of pERK-ERK proteins in Neuro2A cells treated with comp#1 (0.01 μM), comp#2 (0.01 μM) and MEK inhibitor (PD98059) (20 μM) (c–d) mRNA expression level of BDNF in zebrafish larvae treated with compounds (1 μM), with and without MEK inhibitor (PD98059) (20 μM). Values are mean + SEM (*n = *3), each group containing 60 larvae (**e**). *p < 0.005 vs Vehicle-treated, ^#^p < 0.05 vs Compound-treated.

The substantial *in vitro* neurotrophic activities of comp#1 and comp#2 and neurogenic activity of comp#2 in zebrafish and in mouse brain prompted us to probe the molecular mechanisms involved. First of all to test the involvement of ERK pathway, known to be an important player in the neurotrophic activity^6^,^9^ Neuro2A cells were treated with compounds, with or without MEK inhibitor PD98059 (20 μM). The treatment with both comp#1 (0.01 μM) and comp#2 (0.01 μM) exhibited significant increase in the neurite length, i.e. neurotrophic action, only when the inhibitor was not present in the media. Comp#1 and comp#2 failed to act as neurotrophic molecules when the cells were pre-treated with the MEK inhibitor, as shown by the reduced neurite length (Fig. 6a,b).

Similarly, comp#1 (0.01 μM) and #2 (0.01 μM) treatment alone showed increase in pERK protein expression levels. The level of pERK in cells was significantly attenuated to the level observed in the vehicle-treated group when comp#1 and #2 treatment was done in the presence of MEK inhibitor (Fig. 6c,d).

Phosphatidylinositol-3 kinase (PI3)-AKT pathway has also shown to be involved in the action of neurotrophic factors^6^ and so the involvement of this pathway was evaluated in the neurotrophic action of the compounds. The data suggest that comp#1 (0.01 μM) and #2 (0.01 μM) treatment did not result in the attenuation of pAKT protein level when cells were pre-treated with the PI3 kinase inhibitor (LY294002) (Supplementary Fig. 2). The analysis of all immunoblot results suggests that both the compounds appear to act through MEK-ERK pathway and not via PI3-AKT pathway.

Further, to identify the involvement of downstream signaling molecules of the MEK-ERK pathway, we examined the expression of BDNF in zebrafish larvae upon treatment with 1 μM solution of comp#1 and #2, as the neurotrophic action has been shown to be mediated by one of the critical downstream targets BDNF. Comp#1 and comp#2 induced significant increase in BDNF gene expression when compared to the level seen in the vehicle treatment, as evident by the quantitative real time PCR analysis (Fig. 6e). However, the expression of BDNF was downregulated in comp#1 and comp#2 -treated larvae which were pre-treated with MEK inhibitor (Fig. 6e).

### The neurotrophic activity of compounds is mediated via BDNF receptor TrkB

**Figure 7:**
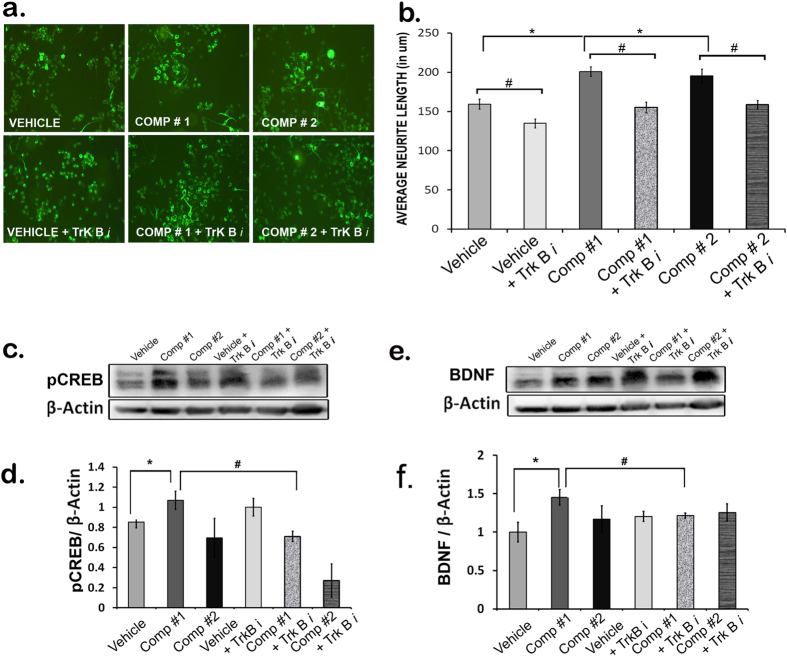
Mechanistic studies of compounds with TrkB inhibitor. Morphometric analysis of neurite outgrowth of differentiated Neuro2a cells treated with comp#1 (0.01 μM), comp#2 (0.01 μM) and vehicle (DMSO), pre-treated (with or without TrkB inhibitor (ANA-12) (10 μM) (a–b). Immunoblot data showing pCREB (**c,d**) and BDNF (**e,f**) expressions in the mouse pup organotypic hippocampal slice culture treated with comp#1 (50 μM), comp#2 (50 μM) and DMSO (pre-treated with or without TrkB inhibitor (ANA-12) (25 μM); n = 4; *p < 0.05 vs Vehicle-treated, ^#^p < 0.05 vs Compound-treated.

Once it was discovered that the compounds increase the transcript level of the neurotrophic factor Bdnf *in vivo* in zebra fish larvae further investigation was done to find out whether this action is mediated through the activation of BDNF receptor TrkB. So, once again we started with *in vitro* system, N2A cells, which upon the treatment with Comp#1 and #2 (0.01 μM) showed substantial increase in the neurotrophic activity as compared to that of the vehicle treatment. N2A cells when pre-treated with ANA-12 (10 μM), a TrkB inhibitor, attenuated the compound-induced neurite growth to significant level (Fig. 7a,b). Next, we tested whether TrkB mediates the neurotrophic action of compounds *in vivo*. Comp#1, but not Comp#2 (50 μM), when added to the mouse acute hippocampal slice culture led to significant increase in the neurotrophic factor BDNF at protein level, as evident in the immunoblot (Fig. 7c,d) and pre-treatment with TrkB inhibitior ANA-12 (25 μM) resulted in the failure of induction of BDNF by comp#1 (Fig. 7c,d).

Since BDNF is the most downstream molecule in TrkB-MEK-ERK-CREB signaling pathway and regulated by the transcription factor CREB, blocking of TrkB receptor should prevent the compound-induced CREB activation measured by pCREB level. With this idea, the level of expression of pCREB and BDNF proteins was assessed in mouse hippocampal slice culture experiment. As expected, pre-treatment with TrkB inhibitor ANA-12 (25 μM) prevented comp#1 (but not comp#2) -induced pCREB and BDNF expressions (Fig. 7c,d). Interestingly, similar observations were found in another *in vivo* experiment using zebrafish embryos where, comp#1 (but not comp#2) induced the transcription of CREB, the transcription factor that bind to the promoter and regulates the expression of BDNF gene (Supplementary Fig. 3).

### Induction of histone H3Acetylation in comp#1 and comp#2-treated IMR-32 cell line

Histone deacetylase (HDAC) inhibitors are epigenetic modulators known to increase histone acetylation by blocking HDACs and opening up of chromatin around the regulatory regions of genes thus causing transcriptional activation, increased neurogenesis and neurotrophic activity *in vivo*. They also play neuroprotective role in a number of neurological and psychiatric disorder models^9^,^10^. So, the possible role of our compounds as epigenetic modulator, in particular as inducer of histone acetylation was also tested. Comp#1 (1 μM) and comp#2 (0.1 μM) induced H3 acetylation in human neuroblastoma cell line IMR-32, compared to the level in vehicle (1% DMSO)-treated cells (Supplementary Fig. 4). The immunofluorescence data suggest that both the compounds were able to increase histone H3 acetylation, not histone H4 acetylation, at very low dose (especially comp#2) similar to the classical HDAC inhibitor sodium butyrate (Supplementary Fig. 4). However, this can be an indirect epigenetic modulatory role of these compounds via activating CREB, known to be an inducer of histone acetylation and transcription activation, as HDAC inhibitor assay was not performed.

## Discussion

Neurodegenerative and neuropsychiatric disorders are major debilitating brain disorders where loss of neurons or neuronal connections and diminished neurogenesis are quite prominent. There are potential molecules that attenuate the neuronal loss and rescue neurogenesis showing promising therapeutic efficacy in treating these disorders^11^,^12^. However, there is still a dearth of drugs as most of the potential small molecules failed rigors of preclinical and clinical trials faced with limitations like unfavorable synthesis, inadequate bioavailability and efficacy to maintain neuroprotection and boost neurogenesis in *in vivo* disease models^11^. So, this investigation was done as an endeavor to develop the potential CNS therapeutic molecules. These two novel natural product (Paecilomycine A)-inspired scaffolds, comp#1 and comp#2, showed potent *in vitro* neurotrophic activity. Additionally, one of these, comp#2, shows *ex-vivo* neurogenic activity in mouse hippocampal neural progenitors cell culture. Comp#2 also appears to be good proneurogenic molecule *in vivo*, as it enhances significant level of neurogenesis in zebrafish and Nestin-GFP mouse brain. On the other hand, comp#1, which has potent neurotrophic but little neurogenic action, on acute administration showed remarkable neuroprotection in global ischemic mouse model (BCCAO).

So, what could be the difference at the molecular level in the differential behavior of these two compounds? The molecular data suggest that the robust neurotrophic action of comp#1 can be attributed to its ability to enhance the transcript level of most of the genes that code for neurotophic factors such as BDNF, GDNF, NT3 and NGF (Fig. 4c). Comp#2 is also neurotrophic but not as good as comp#1 as it failed to increase the mRNA level of NGF, rather it attenuated its level compared to the vehicle treatment (Fig. 4c). In addition, the level of increase in mRNA level of GDNF and NT3 observed in embryos after the comp#2 treatment was not as high as in comp#1 treatment (Fig. 4c). This could explain the differential activity of comp#1 (more neurotrophic) and comp#2 (more neurogenic, less neurotrophic), in spite of the fact that BDNF protein expression levels were prominently high in Neuro2a cells treated with both comp#1 and comp#2, like honokiol used as a positive control, compared to vehicle-treated cells (Fig. 4a,b).

The neurotrophic activity is reported to be mediated through MEK-ERK and PI3-AKT signaling pathways, downstream to the activation of neurotrophin receptors^13^,^14^,^15^. The findings that comp#1 and #2 cause reduction in neurite length and pERK protein level in Neuro2A cells upon pre-treatment with MEK inhibitor (PD98059), suggests that the neurotrophic action of these molecules is mediated through the MEK-ERK pathway. Further, the downstream target molecule (BDNF expression) activated by the compound induced MEK-ERK-CREB pathway was assessed^10^. The level of BDNF mRNA was attenuated when the MEK inhibitor was administered to zebrafish embryos prior to the treatment with compounds (Fig. 6e), suggestive of the involvement of this classical neurotrophin signaling pathway. However, there was no change in the level of pAKT after the treatment with compounds in presence of PI3K inhibitor (an inhibitor of AKT pathway) (Supplementary Fig. 2), ruling out the involvement of AKT signaling pathway in the neurotrophic and neurogenic action of compounds.

Finally, studies were done to find out whether the compound-induced neurotrophic activity is mediated by the receptor through which BDNF acts, i.e. TrkB receptor, using *in vitro* (N2A cells), *ex vivo* (mouse organotypic hippocampal slice culture) and *in vivo* (Zebra fish embryo) systems. The outcome of these experiments (Fig. 7a–d) confirms that the neurotrophic activity of comp#1 is through TrkB receptor in both *in vitro* and *in vivo models.* Comp#2, on the other hand, seems to work as neurotrophic molecule via TrkB receptor only in *in vitro* condition (Fig. 7a,b), similar to what has been reported earlier for other neurotrophic molecules^16^.

Recent research implicates the role of histone acetylation/deacetylation, one of many epigenetic regulatory mechanisms, in the etiopathology of neuropsychiatric disorders, including neurodegenerative diseases^9^,^17^. Histone deacetylase (HDAC) inhibitors cause increase in histone acetylation by inhibiting HDACs resulting in opening up of chromatin around the regulatory regions of genes thus causing transcriptional activation, increased neurogenesis and neurotrophic activity *in vivo*^18^. They also play neuroprotective role in animal models of a number of neurological and psychiatric disorders^18^. So, the role of these compounds as epigenetic modulator (such as HDAC inhibitor) was tested and it seems that both the compounds were able to increase histone H3 acetylation at very low dose (especially comp#2), similar to the classical HDAC inhibitor sodium butyrate (see Supplementary Fig. 4). However, whether the induction in H3 acetylation is direct effect of the compounds on HDACs (as inhibitor) or indirect effect of compound-induced pCREB binding on the target gene promoters resulting in enhanced recruitment of histone acetyl transferases and thus increased acetylation of histone, will be interesting to look into.

In summary, our study reports two small molecules, comp#1 and comp#2, synthesized by Diverted Organic Synthesis (DOS) with less structural complexity than Paecilomycine A (natural parent compound), having potent neuroactivity at low concentration. Comp#1 appears to provide neuroprotection in stroke model and can be taken to the next level of therapeutic development. Comp#2 failed to provide neuroprotection in this model in acute single dose treatment. Comp#2 might show neuroprotection in this or other model if administered chronically, as adult neurogenesis is slower process. Thus, further studies are needed to analyze the efficacy of chronic treatment with comp#2 in brain disorders where the neurogenesis is severely affected. The stage is now set for evaluating the efficacy of these compounds and their analogues in various other rodent models of neurological and psychiatric disorders, including cognitive disorders, where neurotrophic and/or neurogenic activities are severely compromised.

## Materials and Methods

### Cell Culture

Mouse Neuroblastoma cells (Neuro2a cells) were obtained from the American Type Culture Collection (ATCC) and were grown in Eagle’s Minimum Essential Medium (EMEM) supplemented with 10% fetal bovine serum, 1% of 100X antibiotic pencillin-streptomycin (Sigma) at 37 °C in 5% CO_2_/95% air. Neuro2A cells were seeded at 10,000 cells/cm^2^, and after 24 h the medium was replaced with the serum-deprived medium (EMEM+0.1% FBS) with and without compounds. Compounds were diluted in DMSO to different concentrations. The final DMSO concentration in each sample was 1% and this concentration did not affect cell growth or viability to any observable extent.

### Animals

For *ex vivo* neurosphere assay, 2-3 days old C57bl/6 pups were used. For *in vivo* assays Zebrafish embryos of three days post-fertilization (3dpf), C57bl/6 adult male mice and Nestin-GFP transgenic mice around 10 wks old, were used as required in various experiments. The wild type strain of zebrafish was maintained in a controlled environment with 14 h light/10 h dark cycle at 28 ± 1 °C. Embryos were collected after natural spawning, staged according to the standard criteria and raised synchronously at 28.5 °C in E3 medium (5 mM NaCl, 0.17 mM KCl, 0.4 mM CaCl_2_, and 0.16 mM MgSO_4_; pH 7.4)^19^. Mice were maintained in Animal House facility of Centre for Cellular and Molecular Biology (CCMB), Hyderabad, India. The animal room was maintained at 25 °C with a 12 h:12 h light-dark cycle (lights off between 18.0 h to 6.0 h). Food and water were available to the animals *ad libitum*. All animal procedures were carried out in “accordance” with the approved guidelines of the Institutional Animal Ethics Committee of the Centre for Cellular and Molecular Biology (CCMB), Hyderabad, India. The mouse protocol no. was IAEC/CCMB/29/2013-14 under institutional registration no.#20/GO/RBi/S/99/CPCSEA and zebrafish protocol no. was IICT/CB/SC/281114/30 under registration no.# 97/1999/CPCSEA. These protocols strictly follows the guidelines of Committee for the Purpose of Control and Supervision of Experiments on Animals (CPCSEA), Government of India for animal welfare.

### Neurosphere assay

Mouse hippocampal neural progenitor/stem cell (NSCs/NPCs) cultures were carried out according to^20^. In brief, C57bl/6 mouse pups (2-3 days old) were taken, their heads decapitated, washed and hippocampi dissected out in ice-cold incomplete Dulbecco’s modified Eagle’s medium. Tissues were finely minced, digested in 0.025% of Trypsin-EDTA for 7 mins at 37 °C and the reaction was arrested by the addition of equal amount of warm 0.014% w/v Trypsin inhibitor containing 1 mg/ml of DNase-1. The tissue chunks were triturated vigorously to produce single cell suspension, which was passed through 40 µM nylon mesh and then spun down. The pellet was resuspended in Neurocult basal medium with proliferation supplements (Stem Cell Technologies), 0.02% BSA (Sigma), 10 ng/mL bFGF (Sigma), 20 ng/mL EGF (Calbiochem), and 0.04 mg/mL Heparin (Sigma), by gentle trituration. The cells were plated at a density of 1000 cells/well in 96-well plate and comp#1, comp#2, vehicle was added after 24 hrs of plating. The number of neurospheres (>100 µm) obtained was counted after 5-6 days in culture. For each drug dose, neurospheres from 5 wells were counted. The experiment was repeated thrice.

### Neurogenesis in zebrafish and mouse

For BrdU labelling, 4 days post fertilisation (d.p.f.) zebrafish larvae were incubated in 10 mM BrdU (Sigma) in E3 media for 2 hours. Following fixation in 4% PFA, larvae were processed for immunofluorescence study with mouse anti-BrdU antibody (1:500) followed by Alexa 488-conjugated secondary antibody (1:1000) were used to reveal BrdU in GFP + cells. The images were taken by stereomicroscope (Leica).

The Nestin-GFP transgenic mice as described previously^21^ were used to address the effect of Comp#2 on hippocampal neuronal proliferation. The animals were given an injection of Comp#2 (1 mg/kg) once daily by i.p injection for a period of 7 days. Another group of mice was used as control and was given an i.p injection of saline for the same period. All the animals were also injected once with BrdU (50 mg/Kg) after 30 mins of drug and vehicle injection for 7 days. After 24 hrs of the last dose the animals were sacrificed, brain were taken out, fixed in 4% paraformaldehyde overnight on the rotator and finally cryopreserved in 20% glycerol for 24 h, before cryo-sectioning. Serial coronal brain sections (30 μm thick) through hippocampus were cut using cryostat (Leica CM 1950, Germany) and processed for the immunofluoroscence staining procedure as described earlier^9^. Briefly, a series of sections were blocked in 10% normal horse serum prepared in 0.3% Triton-X for 2 hrs at room temp, followed by overnight incubation in anti-mouse GFP (1:100, Santa Cruz) at 4 °C. Next day, after washes the sections were incubated in goat anti-mouse IgG-FITC (1:1000, Santa Cruz) for 45 mins at room temp and finally mounted in Vectashield medium (Vector labs) with DAPI. GFP positive cells were quantified by stereological cell counting method^22^.

For BrdU immunostaining, SuperPicture IHC detection kit (Invitrogen) was used as previously reported^12^. In short, the sections were pre-treated with 50% formamide in 2X SSC for 2 hrs at 65 °C, to denature the DNA. After washes, acid treatment with 2N HCl was given for 30 mins at 37 °C and then neutralized with 0.1 M Boric acid (p.H 8.5). Peroxide quenching was done with 3% H_2_O_2_ for 15 mins at room temperature. The sections were then blocked in 10% horse serum for 2 hrs at RT, followed by overnight incubation in primary antibody (anti-Mouse BrdU, Calbiochem, 1:500). Next day, the sections were incubated in secondary antibody (HRP Polymer) for 45 mins and finally developed with diaminobenzidine (DAB) before mounting in DPX.

### Ischemic stroke and the treatment with compounds

Global ischemia was induced surgically by Bilateral Common Carotid Artery occlusion (BCCAO) in C57bl/6 male adult mice of 10 wks old. The animals were anaesthetized with an intraperitoneal injection of ketamine/xylazine cocktail (100 mg/kg). BCCAO was performed according to previous report^22^ with appropriate modification. In brief, a 2 cm long incision was made on both the left and right ventral neck region. Common carotid arteries were exposed carefully without disturbing other nerve and arteries, and were clamped using non-traumatic bulldog clip for 5-7 minutes. Reperfusion was done after 5-7 min of occlusion and animals were returned to their home cage. After 24hrs of BCCAO, brain was collected for Golgi-Cox histochemical staining. Fifteen minutes before ischemia the compounds were injected (10 mg/Kg bodyweight) and after 24 hours Brains were collected for Golgi-Cox Staining.

### Golgi-Cox Staining

After 24 hours of reperfusion following BCCAO the mice were sacrificed by cervical dislocation and Golgi-Cox staining was done as described by previous researchers^23^,^24^,^25^. In short, brain was immediately dropped into Golgi-Cox solution (diluted from a freshly prepared solution containing 5% potassium dichromate and 5% mercuric chloride in 1:1) and left in a dark place for 3-7 days. After cryoprotection in 30% sucrose solution, 50 μm sections were cut using vibratome (OTS-4500, Harvard Apparatus, USA), dropped in a chamber filled with 6% sucrose solution. Sections were rinsed in double distilled water (DDW) and transferred into ammonia solution (ammonium hydroxide: DDW 2:1) for 5-7 min. The sections were then incubated in a solution containing 0.5% sodium thiosulfate plus 0.2% sodium metabisulfite for 7-10 minutes after rinsed in DDW. The sections were finally mounted carefully on 2% gelatin coated slides after a thorough rinse with DDW, dehydrated, cleared in isopropyl alcohol and cover slipped with DPX mounting media. For analyses, 25 dendritic segments (50 μm each) were randomly selected from each group and measured for spine density using Image J software.

### Immunocytochemistry

For immunocytochemistry, cells after 24 hours in culture with various treatments, were processed for the immunostaining as previously reported^14^. Briefly, cells were fixed with 4% paraformaldehyde at room temperature for 15 minutes, permeabilization was done with 0.5% TritonX-100 and 0.05%Tween20 in 1XPBS, followed by incubation in blocking buffer (2% bovine serum albumin + 0.1% Triton X-100 in 1XPBS) for 2 hours at room temperature. Primary antibody against b III tubulin (1:200, Millipore) was used to visualize neurons. Samples were incubated with primary antibody in blocking buffer overnight at 4 °C. Samples were washed with PBST (PBS with 0.1%Tween20) and incubated with goat anti-mouse IgG conjugated to AlexaFlour 488 (1:400, Molecular Probes). Images were captured using a MoticAE31 microscope.

### Immunoblotting

Cells in wells after incubation with compounds, vehicle, with and without inhibitor treatment were washed with 1XPBS and harvested in 1X Laemmli buffer. Protein estimation was done with amido black method and equal amount of protein was loaded onto 12% SDS-PAGE gel. Blocking was done for 1 h at room temperature, followed by incubation with primary antibodies BDNF (1:1000) (Millipore), ERK (1:2000) (CST), pCREB (1:1000) (Millipore), Actin (1:5000) (Sigma), pAKT (1:500) (CST) and AKT (1:1000) (CST) at 4 °C overnight, while pERK (1:3000) (Abcam) was incubated for 2 h at room temperature. Incubation in secondary antibody Anti Rabbit (1:5000), Anti mouse (1:10000) was done at room temperature for 2 h. The blots were developed with Vilber Lourmat chemdoc instrument using super signal west dura luminal/enhancer solution (Thermo).

### RNA Isolation and Gene Expression

The total RNA was isolated from each set of treated groups containing (n = 60) larvae using TRIzol Reagent (Invitrogen) according to the manufacturer’s protocol. cDNA was synthesized using RevertAid H Minus First Strand cDNA Synthesis Kit following the manufacturer’s instructions. Primer sequences used for *in vivo* are available upon request. Quantitative real-time PCR was performed in triplicate by using SYBR Green PCR Master Mix Detection System (Applied Biosystems). The mRNA level was normalized by housekeeping gene β-actin. Relative gene expression analysis was performed for genes that code for BDNF, GDNF, NT3, NGF and CREB.

### Inhibitor Assays

In Neuro2A cells: 10,000 cells/cm^2^ were plated on with or without cover slips in culture dish. After 24 h of incubation, cells were treated with PD98059 (MEK1/2 inhibitor) (20 μM), LY294002 (Phosphatidylinositol-3-kinase inhibitor) (20 μM) and ANA-12 (TrkB inhibitor) (10 μM). After 1 h of incubation with inhibitors cells were treated with compounds and allowed for 4 h and 24 h of incubation. Cells were harvested and used for Western blotting and immunocytochemistry.

In Zebrafish: Embryos of 3dpf zebrafish were treated with compounds (pre-treated with and without MEK inhibitor (PD98059), TrkB inhibitor (ANA-12). After 24 h of the treatment the embryos were pooled for RNA isolation followed by Quantitative real-time PCR.

In mouse organotypic slice culture: As previously reported^26^ for organotypic slice culture 5-7 postnatal day old C57bl/6 pups were taken and decapitated and brains were quickly dissected out in ice-cold artificial cerebrospinal fluid (aCSF)[Soln A: NaCl- 8.66 g, KCl- 0.224 g, CaCl_2_.2H_2_O- 0.206, MgCl_2_.6H_2_O- 0.163 g dissolved in 500 mL of sterile pyrogen free water; Soln B: Na_2_HPO_4_.7H_2_O- 0.214 g, NaH_2_PO_4_.H_2_O- 0.027 g dissolved in 500 mL of sterile pyrogen free water; both the solutions A and B were dissolved in 1:1 ratio just before use and refrigerated at 4 °C immediately before use]. After giving a wash in fresh cold aCSF, each brain was gently placed into the brain matrix and 1 mm brain slices, through hippocampus were obtained. Each slice was then transferred to a 12-well tissue culture plate containing 1.5 mL of pre-warmed and well-aerated aCSF. Then the half of the total number of slices was treated with 25 µM of TrkB inhibitor (**ANA-12** SIGMA-SML0209-5MG) and DMSO as vehicle. The plate was returned to the tissue culture incubator (37 °C, 5% CO_2,_ 95% O_2_) and incubated for 30 mins. After half an hour, 50 µM of compounds (Comp #1 & Comp #2) were treated to slices with or without inhibitor treatment and incubated inside the tissue culture incubator for next 2 hours. After 2 hours, the slices were given a wash with ice-cold aCSF and two-three random punches (excluding pons area) were obtained with a 12-gauge needle from each slice. 50 µl of the 1X lamaelli buffer was added to each tube and homogenized by sonication in a Bioruptor and boiled at 100 °C for 10 mins. After estimating the protein content by Amido black method SDS-PAGE was run with equal protein lysate loading in each well and western blotting was performed with anti-BDNF, anti pCREB and anti-ß-Actin antibodies.

### Histone acetylation Assay

Human neuroblastoma cells IMR32 were plated at 10,000 cells/cm^2^ in DMEM (10%FBS). After 24 h, cells were treated with compounds (sodium butyrate, comp#1 and comp#2) and vehicle and 24 h later were processed for immunostaining as described above for the acetylated H3, acetylated H4 proteins and DAPI.

### Statistical Analysis

The results were expressed as means ± S.E.M. The data were subjected to statistical analysis using Student’s t-test. A value of P < 0.05 was accepted as the level of significance.

## Additional Information

**How to cite this article**: Chakravarty, S. *et al.* A novel natural product inspired scaffold with robust neurotrophic, neurogenic and neuroprotective action. *Sci. Rep.* **5**, 14134; doi: 10.1038/srep14134 (2015).

## Supplementary Material

Supplementary Information
