# Supplementary material for: A novel natural product inspired scaffold with robust neurotrophic, neurogenic and neuroprotective action
Source: Sci Rep. 2015 Sep 21;5:14134. doi: 10.1038/srep14134 (PMC4585672; doi:10.1038/srep14134)
Supplement: Supplementary Information [file srep14134-s1.pdf]

A novel natural product inspired scaffold with robust neurotrophic, neurogenic and neuroprotective action

Sumana Chakravarty[1]*, Swati Maitra[1#], R Gajendra Reddy[1#], Tapatee Das[1#], Priya Jhelum[1], Scherazad Kootar[2], Wenson D Rajan[2], Anumita Samanta[1], Ramesh Samineni[3], Srihari Pabbaraja [3], Steven G Kernie[4], Goverdhan Mehta[5], Arvind Kumar[2]

[1]Chemical Biology and [3]Natural Products Chemistry, CSIR- Indian Institute of Chemical Technology, Tarnaka, Uppal Road, Hyderabad 500007, India.

[2]CSIR- Centre for Cellular and Molecular Biology, Habsiguda, Uppal Road, Hyderabad 500007, India.

[4]Department of Pediatrics and Department of Pathology and Cell Biology, College of Physicians and Surgeons, Columbia University, New York, NY 10032, USA.

[5]School of Chemistry, University of Hyderabad, Hyderabad-500046, India.

[#] These authors contributed equally to this work.

Suppl fig 1.

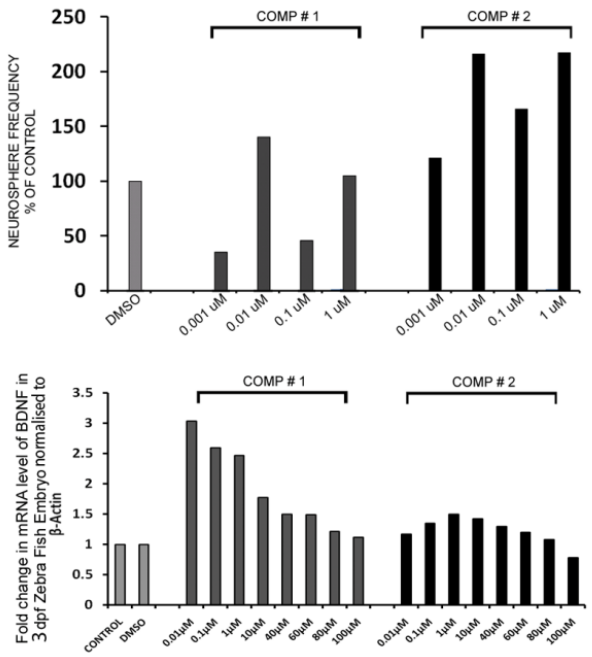

Supplementary fig.1: Dose titration for ex vivo and in vivo experiments. Upper panel showed the neurosphere assay by using mouse hippocampal precursor cell populations subjected to different concentrations of the compounds from [0.001µM to 1.0 µM]; whereas lower panel showed Bdnf gene expression level in Zebrafish embryos treated with different concentrations of the compounds [0.01µM to 100 µM]. Please note that the most optimum concentrations [0.01 µM for mouse primary culture, 1.0 µM for zebrafish emryo experiments] were selected for main experiment. To decide the exact dose, similar methods were followed in other experiments too.

Suppl fig 2.

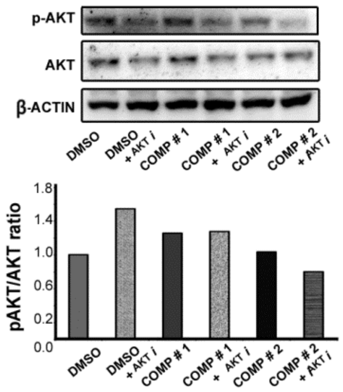

Supplementary fig.2: Immunoblot for pAKT and AKT. Expression levels of p-AKT/AKT in Neuro2A cells treated with comp #1, #2 (0.01µM), pretreated with or without AKT inhibitor (LY294002) (20µM).

Suppl fig 3.

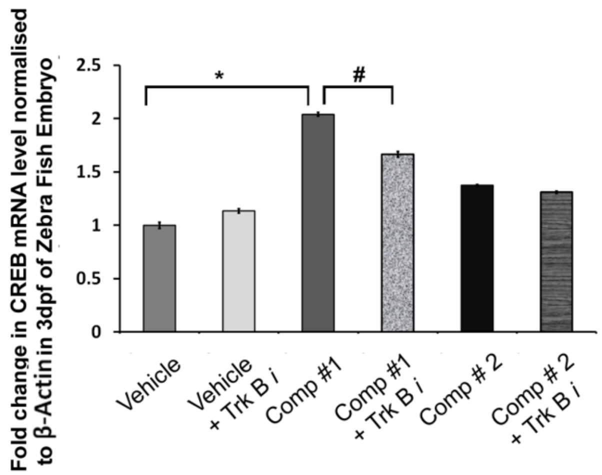

Supplementary fig.3: RT-PCR data for Creb upon Trk B inhibitor treatment. Shown here is the graphical representation for the mRNA expression level of Creb normalized with β-Actin in 3 dpf of Zebrafish embryo upon treatment with/without TrK B inhibitor ANA-12.

Suppl fig 4.

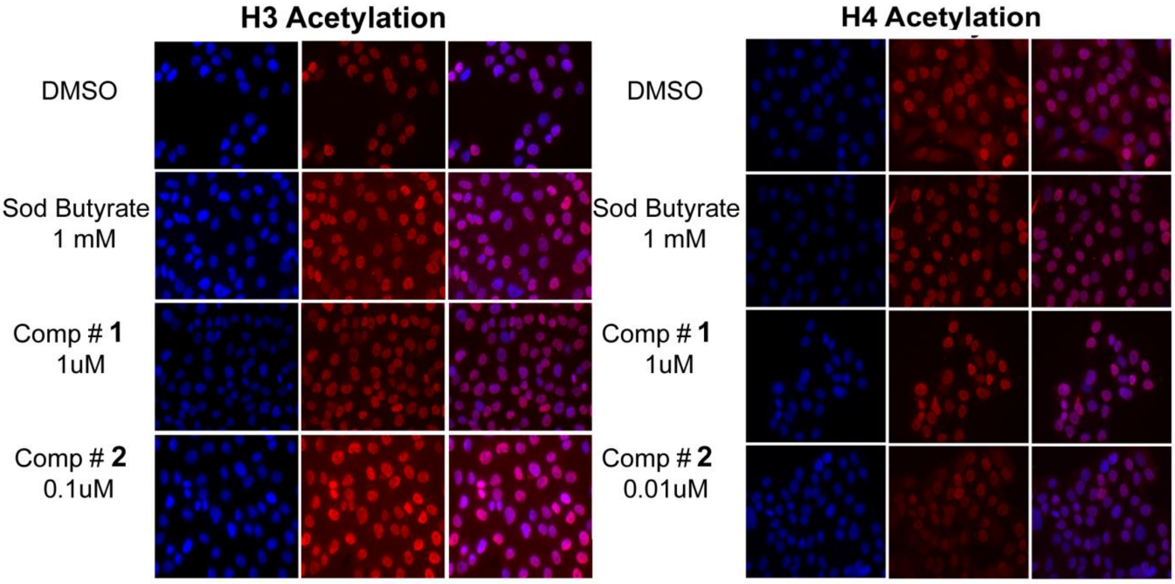

Supplementary fig.4: Immunocytochemistry for acetyl H3, H4 and DAPI. Shown are the representative images for Acetyl H3, H4 and DAPI staining in IMR-32 cells treated with Vehicle (DMSO 1%), Sodium butyrate (1mM), Comp#1 (1µM), Comp#2 (0.1, 0.01µM).
